# Virtual Reality for Anxiety and Pain Management in Lower Limb Orthopedic Surgery Under Spinal Anesthesia: A Randomized Controlled Trial

**DOI:** 10.1002/hsr2.73146

**Published:** 2026-09-24

**Authors:** Fedi Ben Dhaou, Yosra Mejdoub, Maroua Trigui, Emna Mziou, Amal Ayedi, Meriam Boussarsar, Mariem Keskes, Yassine Maktouf, Fares Mezghani, Mohamed Seddik Regaieg, Tarek Bardaa, Chams Elhouda Ben Dhaou, Hichem Cheikhrouhou, Imen Zouche

**Affiliations:** ^1^ Anesthesiology and Intensive Care Department Habib Bourguiba University Hospital Sfax Tunisia; ^2^ Faculty of Medicine University of Sfax Sfax Tunisia; ^3^ Preventive Medicine and Hospital Hygiene Department Habib Bourguiba University Hospital Sfax Tunisia; ^4^ Anesthesiology and Intensive Care Department Military University Hospital Sfax Tunisia; ^5^ Orthopedic Surgery and Traumatology Department Habib Bourguiba University Hospital Sfax Tunisia; ^6^ English Department Izdihar Elementary School Sfax Tunisia

**Keywords:** anxiety, lower limb surgery, pain management, perioperative care, spinal anesthesia, virtual reality

## Abstract

**Background and Aims:**

Patients undergoing lower limb orthopedic surgery under spinal anesthesia often experience significant perioperative anxiety and discomfort. Virtual reality (VR) has emerged as a promising non‐pharmacological intervention for anxiety and pain management. This study aimed to investigate the efficacy of VR as a complementary method for improving perioperative anxiety and pain management in patients undergoing lower limb orthopedic surgery under spinal anesthesia.

**Methods:**

This prospective, single‐center, randomized, single‐blind trial included two groups: a VR group, receiving perioperative VR exposure, and a control group with standard care. The primary outcome was perioperative anxiety reduction measured using the State‐Trait Anxiety Inventory‐6 (STAI‐6) from preoperative period to after surgery. Secondary outcomes included pain intensity, sedative and analgesic medication consumption, hemodynamic stability, postoperative complications, and satisfaction of patients, anesthesia, and surgical teams. A total of 160 patients were randomized into 2 groups, of whom 60 per group were included in the final analysis. The trial was registered with the Pan African Clinical Trial Registry (PACTR202507660086282) and ClinicalTrials.gov (NCT07134764).

**Results:**

The VR group demonstrated significantly lower postoperative anxiety levels compared to controls, with intense anxiety occurring less frequently (25% vs. 50%, *p* = 0.005). STAI‐6 scores showed marked reduction in the VR group (36.5 ± 7.92 vs. 46.72 ± 2.58, *p* < 0.001). Midazolam consumption was significantly reduced in the VR group (20% vs. 53.3%, *p* = 0.001). The VR group experienced significantly fewer postoperative complications including nausea (18.3% vs. 36.7%, *p* = 0.025) and headache (16.7% vs. 35%, *p* = 0.002). Patient satisfaction was significantly higher in the VR group, with dissatisfaction rates dramatically lower (3.3%vs.43.3%, *p* < 0.001). Both anesthesia and surgical teams reported higher satisfaction when managing VR group patients.

**Conclusion:**

VR distraction significantly reduces perioperative anxiety, decreases sedative medication requirements, reduces postoperative nausea and headaches, and improves satisfaction.

## 1 Introduction

Lower limb orthopedic surgery is one of the most common surgical procedures performed to treat degenerative, traumatic, and congenital conditions [1, 2].

Spinal anesthesia has become an effective alternative to general anesthesia. This technique offers several benefits including fewer anesthesia complications, lower costs, and improved patient comfort [3]. Remaining conscious throughout the procedure, and able to “hear and see everything” taking place in the room, is itself a recognized source of distress for patients; this heightened emotional state can, in turn, shape how pain is perceived and how satisfied patients ultimately feel with their care [1]. Managing anxiety and pain during surgery remains a major challenge for patients having lower limb orthopedic surgery under spinal anesthesia [4].

Virtual reality (VR) has emerged as an innovative and promising solution. VR differs fundamentally from simple video viewing or audio listening through several distinct mechanisms. Unlike passive audiovisual distraction, VR creates an immersive three‐dimensional environment that induces a sense of “presence”—the psychological sensation of being transported into the virtual world rather than the physical surgical setting [5, 6]. This immersive presence is achieved through stereoscopic visuals, head tracking that responds to user movements, and spatial audio, which collectively engage multiple sensory channels simultaneously and create a coherent perceptual experience that isolates patients from anxiety‐provoking environmental stimuli in the operating room (OR) [7, 8].

The anxiolytic effects of VR are theorized to operate through several VR‐specific mechanisms. First, the high degree of attentional engagement required to interact with and navigate the virtual environment consumes substantial cognitive resources, leaving fewer attentional resources available for processing pain and anxiety‐related stimuli—a phenomenon supported by gate control theory and cognitive load theory. Second, VR's immersive nature creates sensory isolation from the OR environment, effectively blocking visual and auditory cues (surgical instruments, medical staff conversations, procedural sounds) that typically trigger anticipatory anxiety. Third, the sense of embodied presence in VR—where patients feel physically located within the virtual space—activates different neural pathways compared to passive observation of a two‐dimensional screen, engaging areas associated with spatial navigation and emotional regulation. Finally, VR provides users with a sense of control and agency within the virtual environment, which may enhance self‐efficacy and reduce feelings of helplessness commonly experienced during surgical procedures [7, 9, 10].

In contrast, conventional video or audio distraction provides only passive sensory input without immersion, presence, or interactive engagement, and fails to effectively isolate patients from the OR environment. Previous studies comparing VR to video distraction have demonstrated superior anxiety reduction with VR, supporting the hypothesis that immersive, interactive technology provides benefits beyond simple audiovisual distraction [11].

This study aims to investigate the efficacy of VR as a complementary method to standard perioperative care in patients undergoing lower limb orthopedic surgery under spinal anesthesia.

The primary outcome was the reduction in anxiety measured using the 6‐item State‐Trait Anxiety Inventory (STAI‐6) during the preoperative period and after surgery.

Secondary objectives included the assessment of perioperative anxiety using other validated anxiety scales, evaluation of perioperative pain intensity, analysis of sedative and analgesic medication consumption, assessment of intraoperative hemodynamic stability, documentation of postoperative complications, and evaluation of patient satisfaction as well as satisfaction of the anesthesia and surgical teams.

## 2 Methods

### 2.1 Study Design

This was a prospective, single‐center, randomized, controlled trial conducted in the Anesthesia and Orthopedic surgery departments, reported in accordance with the CONSORT 2025 guidelines for randomized trials. The study was approved by the Ethics Committee of the Faculty of Medicine of the University of Sfax and the Direction of our University Hospital (approval reference: 51/24), and was registered with the Pan African Clinical Trial Registry (PACTR202507660086282) and ClinicalTrials.gov (NCT07134764) prior to enrollment. The study was conducted in accordance with the ethical standards of the Declaration of Helsinki and its later amendments. All patients participated voluntarily after receiving a detailed explanation of the study objectives, research modalities, and implications of participation, and provided written informed consent to participate and for publication of anonymized data. Collected data were anonymized before analysis to ensure no identifying patient information could be disclosed.

Due to the visible nature of the VR intervention, blinding of participants, clinical staff, and outcome assessors was not feasible. However, to minimize detection bias, statistical analysis was performed by a biostatistician who received de‐identified data with treatment groups coded as Group A and Group B, maintaining analyst blinding until completion of all primary and secondary outcome analyses.

### 2.2 Study Population

For the purposes of this study, lower limb orthopedic surgery was defined as surgical procedures involving the hip, femur, knee, tibia, fibula, ankle, or foot, performed under spinal anesthesia, including fracture fixation and reconstructive procedures. Patients were eligible for inclusion if they were aged between 18 and 65 years, classified as American Society of Anesthesiologists (ASA) class I or II, scheduled for lower limb orthopedic surgery under spinal anesthesia, and provided consent to participate in the study. The upper age limit of 65 years was set per the Ethics Committee, given that patients above this age are more likely to present with age‐related comorbidities (including hearing impairment and frailty), reduced tolerance to sedative and anesthetic medications, a higher risk of perioperative complications, and prolonged PACU stay, all of which could confound the assessment of VR‐related anxiolytic and sedative‐sparing effects.

Patients were not included in the study if they refused participation or presented contraindications to virtual reality, including cognitive or psychiatric disorders, claustrophobia, uncontrolled epilepsy, or visual and/or auditory impairments preventing VR use. Patients were also not included if they had contraindications to Midazolam (known hypersensitivity to benzodiazepines or any product excipient), current use of anxiolytics, and contraindications to regional anesthesia. The latter encompassed hemostatic disorders, shock state or uncompensated hypovolemia, unstable arterial hypertension, decompensated heart failure, severe aortic or mitral stenosis, intracranial hypertension, infection near the puncture site, progressive neurological disease, local anesthetic allergy, and technical difficulties in performing spinal anesthesia.

Patients were excluded from the study in case of spinal anesthesia failure requiring conversion to general anesthesia, occurrence of anesthetic or surgical complications, or VR‐related adverse effects, collectively referred to as cybersickness. These adverse effects included dizziness, ocular or muscular contractions with light stimulation, and signs of discomfort such as eye fatigue, altered vision, disorientation, imbalance, anxiety attack, headache, nausea, or vomiting. Patients in the VR group were continuously monitored by the research coordinator throughout VR exposure for clinical signs of cybersickness; any patient developing such symptoms was withdrawn from VR use and managed according to standard PACU protocols, and their outcome data were excluded from the final per‐protocol analysis.

### 2.3 Sample Size Calculation

Sample size calculation was performed using BioStat TGV software, based on results from the study by Arifin et al. [12] using the six‐item State‐Trait Anxiety Inventory (STAI‐6) to measure anxiety immediately after the surgical procedure. This study reported a mean anxiety score of 34 for the control group and 29.4 for the VR group, with a common standard deviation (SD) estimated at 5.08. Using a two‐tailed type I error (α) set at 5% and statistical power (1−β) of 90%, the calculation determined that 26 patients per group were necessary to detect a statistically significant difference. Adding a dropout rate of 20% to account for potential losses to follow‐up, the final sample size was adjusted to 32 patients per group.

Although the a priori power calculation indicated that a minimum of 32 patients per group (64 total) was required to detect a statistically significant difference in the primary outcome, a final sample of 120 patients (60 per group) was enrolled. This larger sample size reflected several considerations established in the study protocol: (1) enrollment continued for the full duration of the pre‐planned data‐collection period, during which all eligible, consenting patients were enrolled; (2) anticipated attrition from spinal anesthesia failure, conversion to general anesthesia, and other protocol deviations common in this surgical population necessitated a buffer beyond the calculated minimum to ensure an adequate number of evaluable patients per group.

### 2.4 Randomization

Randomization was performed using a computer‐generated sequence with permuted blocks of size 6 to ensure a 1:1 allocation ratio between the VR group and control group. The randomization list was generated by an independent statistician and concealed using sequentially numbered, sealed, opaque envelopes. After obtaining informed consent and completing baseline assessments, the research coordinator opened the next envelope in sequence to reveal the group assignment. Participants were then allocated to either the VR distraction group or the control group. Participants were blinded to the study hypothesis, and outcome assessors measuring anxiety scores were blinded to group allocation.

### 2.5 Interventions

**Figure 1:**
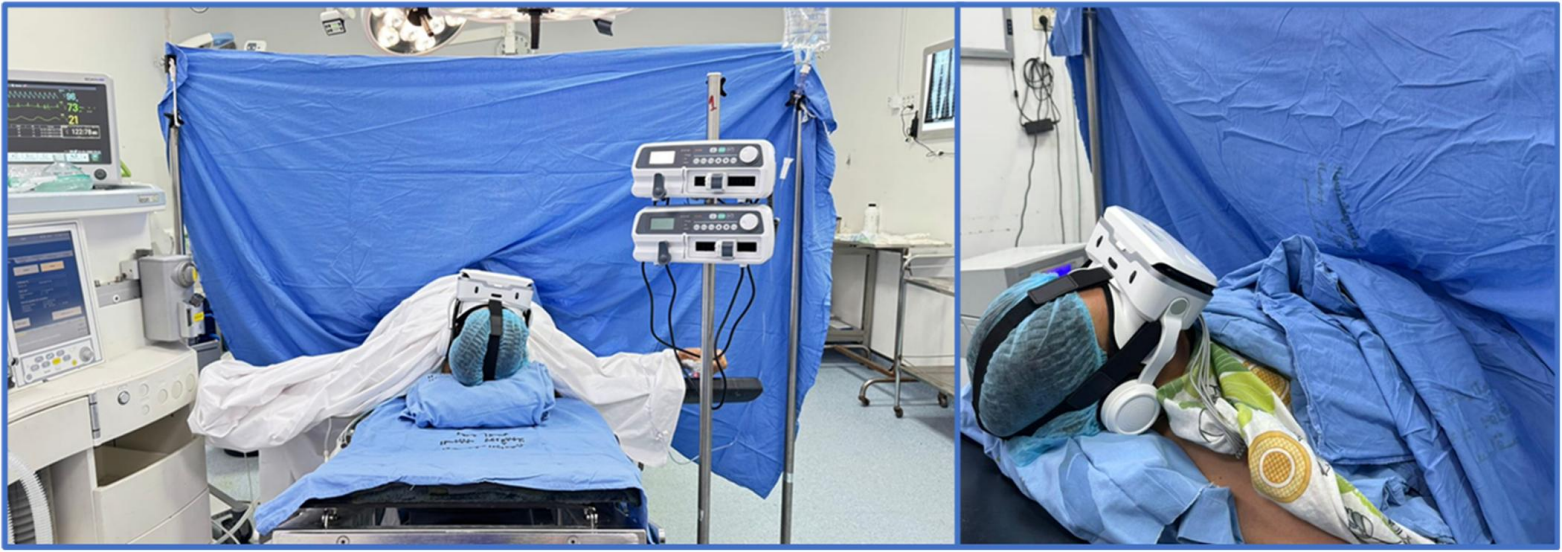
Setup of VR intervention during regional anesthesia in the operating room.

Patients included in the intervention group (VR group) had access to a VR initiation session before surgery, received standard care and received a VR session during surgery (Figure 1). Control group patients received standard care usually administered, without VR use.

### 2.6 Equipment

**Figure 2:**
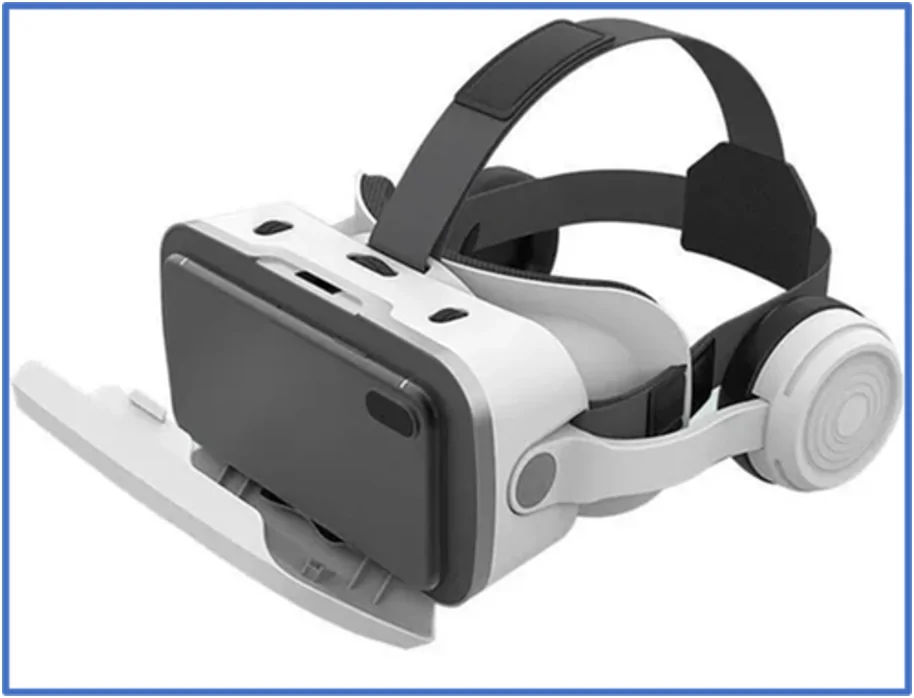
The VR headset with integrated headphones used in the study.

We used a SHINECON® (China) VR headset with the following specifications: high‐definition resin aspherical lenses providing stereoscopic 3D visualization with a 120° field of view, adjustable interpupillary distance ranging from 60 to 70 mm to accommodate individual differences, and built‐in spatial audio headphones connected via 3.5 mm jack to eliminate Bluetooth latency. The headset weighs 350 g and features soft, breathable leather padding for prolonged comfort. A smartphone (Android compatible, minimum 5.5‐inch screen) was inserted into the headset compartment to deliver the VR content (Figure 2).

### 2.7 Virtual Reality Content Description

The VR intervention consisted of immersive nature‐based environments specifically designed for anxiety reduction and relaxation. The video presented a continuous sequence of scenic natural environments: mountain landscapes with snow‐capped peaks, turquoise glacial lakes, and lush green valleys, coastal scenes with dramatic cliffs meeting crystalline blue waters, forest environments with dense vegetation and natural waterways and panoramic mountain vistas with clouds and expansive sky views.

All scenarios featured high‐resolution stereoscopic with binaural spatial audio that changed based on head position and orientation. The experiences were non‐interactive (passive viewing) but incorporated dynamic elements (moving animals, flowing water, swaying vegetation) and responded to head tracking, creating a strong sense of presence and immersion.

The headset's head‐tracking function remained active throughout VR exposure, allowing patients to freely move their head. This was not restricted during surgery, since the surgical field was at the lower limb and head movement did not interfere with it.

The audio track consisted of a single continuous, wordless instrumental melody layered over the natural ambient sounds; the headset itself did not include active noise‐cancellation technology, but the closed‐ear design combined with this continuous audio track served to attenuate operating‐room noise. The same standardized VR video and audio content was shown to every participant in the VR group, with no patient‐to‐patient variation, eliminating content heterogeneity as a potential source of outcome variability. The content was developed and selected by a multidisciplinary panel including the psychiatry department with expertise in relaxation‐based interventions, who reviewed candidate footage for anxiolytic suitability, absence of distressing or disorienting elements, and cultural neutrality prior to the start of the trial.

The video (approximately 120 min per cycle) looped seamlessly and continuously throughout the entire surgical procedure from the moment of spinal anesthesia administration until completion of surgery, ensuring uninterrupted immersive exposure regardless of individual procedural duration (Video S1).

### 2.8 Data Collection

Patient‐related data included sociodemographic, anthropometric, and clinical information, encompassing ASA classification, medical and surgical history, lifestyle habits, and specific history related to spinal anesthesia and postoperative morphine use.

Surgery and spinal anesthesia data included surgical procedure details, and the sequence of surgical steps. Perioperative analgesic and sedative medication consumption was recorded, specifying administration details, quantity, and medication types.

Preoperative anxiety was assessed using three validated instruments: the Visual Analog Scale (VAS), the Amsterdam Preoperative Anxiety and Information Scale (APAIS), and the STAI‐6. Pain intensity was measured with the VAS for pain. VAS scores were categorized as mild‐to‐moderate (1–5) or intense (≥ 6), for consistency with the categories reported in Table 3. For APAIS, global scores > 11/20 indicated high anxiety, while information‐need scores were classified as low (2–4), moderate (5–7), or high (> 7).

Hemodynamic monitoring included systolic and diastolic blood pressure (SBP, DBP), heart rate (HR), and oxygen saturation, recorded at regular intervals. Perioperative monitoring also captured hemodynamic variations and medication use.

Patient, surgical, and anesthesia team satisfaction were evaluated using validated objective criteria [3] rather than simple subjective rating scales. Surgeon and anesthesiologist satisfaction were based on observable clinical events (patient movement frequency, apnea episodes, desaturation events) that can be documented objectively, while patient satisfaction incorporated measurable parameters (surgical recall). These objective observations were then mapped to a four‐level categorical satisfaction scale (dissatisfied, satisfied, extremely satisfied, and an intermediate level) using a predefined scoring rubric based on the number and severity of observed events (Supplement File S2).

In the postoperative unit, the presence of nausea, vomiting, and headache was systematically assessed. Cybersickness (as defined above) was actively monitored throughout the VR session by direct clinical observation and patient self‐report; any occurrence prompted immediate discontinuation of VR use and was recorded.

### 2.9 Study Protocol

Pre‐anesthetic consultation was conducted with standardized data collection. Patients provided informed consent and were familiarized with the VR device and given a brief preview of the standardized VR content upon entering the OR. Anxiety and pain were evaluated at T0 using VAS, STAI‐6, and APAIS scales.

Standard monitoring and a 20 G IV line were established. Anxiety and pain were reassessed at T1. All patients received 500 mL of saline, followed by spinal anesthesia (Hyperbaric 0.5% Bupivacaine 10 mg + Sufentanil 3 µg) at the L3–L4 or L4–L5 level. Patients were positioned semi‐sitting with a 30° inclination and received nasal oxygen at 4 L/min.

Sensory block was evaluated every 2 min using ether‐soaked compresses, and motor block every 5 min using the Bromage scale. After block installation and hemodynamic verification, anxiety and pain were measured at T2, after which the VR group began video viewing.

Hemodynamic parameters (HR, SBP, DBP, and oxygen saturation) were recorded every 10 min. Midazolam (1 mg, maximum) was administered for agitation, HR increase > 20%, or anxiety VAS ≥ 4. Atropine was used for bradycardia (HR < 50 bpm), and ephedrine for hypotension (SBP < 90 mmHg).

At the end of the intervention, anxiety and pain were assessed at T3, along with satisfaction of the patient, surgical team, and anesthesia team. Anxiety and pain levels were subsequently evaluated with VAS at 2 h (T4), 4 h (T5), and 6 h (T6) post‐intervention to monitor their progression and assess the method's effectiveness.

In the Post‐Anesthesia Care Unit (PACU), hemodynamic parameters were continuously monitored, and the presence of nausea, vomiting, or headache was recorded. An analgesic protocol was applied consistently across the OR, PACU, and orthopedic department.

**Figure 3:**
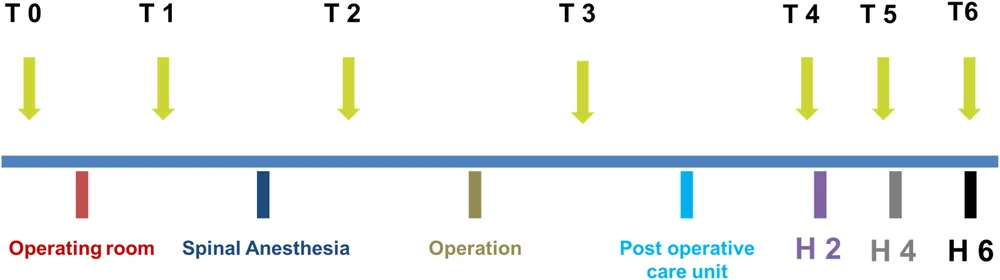
Timeline of assessments at different perioperative stages.

Overall, the study followed a precise timeline, enabling systematic evaluation of anxiety and pain at multiple perioperative stages (Figure 3).

### 2.10 Statistical Analysis

Data were entered, verified, and analyzed using Statistical Package for the Social Sciences (SPSS) version 25. Qualitative variables were described as frequencies (N) and percentages (%). Normality of quantitative variables was verified using the Kolmogorov‐Smirnov normality test. Means ± SD were used to describe normally distributed quantitative variables. Otherwise, medians and interquartile ranges (IQR) were used.

For comparing two percentages, the Chi‐square test was performed or Fisher's test when Chi‐square application conditions were not validated. For comparing multiple percentages, the Chi‐square test was performed. For comparing 2 means, the Student's t‐test was performed if variables were normally distributed or the Mann‐Whitney test otherwise. Statistical significance threshold was set at *p *≤ 0.05. All statistical tests were two‐sided. The primary outcome (STAI‐6 anxiety score) and the pre‐specified secondary outcomes (pain intensity, sedative/analgesic and opioid consumption, hemodynamic parameters, postoperative complications, and satisfaction) were analyzed as defined in the study protocol; no exploratory or post hoc subgroup analyses were performed.

For the primary outcome measures (STAI scores and Anxiety‐VAS), two‐way repeated measures analysis of variance (ANOVA) was conducted with group (VR vs. Control) as the between‐subjects factor and time (measurement points) as the within‐subjects factor. The assumption of sphericity was assessed using Mauchly's test, and when violated, Greenhouse‐Geisser corrections were applied to adjust the degrees of freedom. Significant main effects and interactions were further explored using pairwise comparisons with least significant difference (LSD) adjustment. Effect sizes were reported as partial eta‐squared (η^2^) for ANOVA results.

## 3 Results

**Figure 4:**
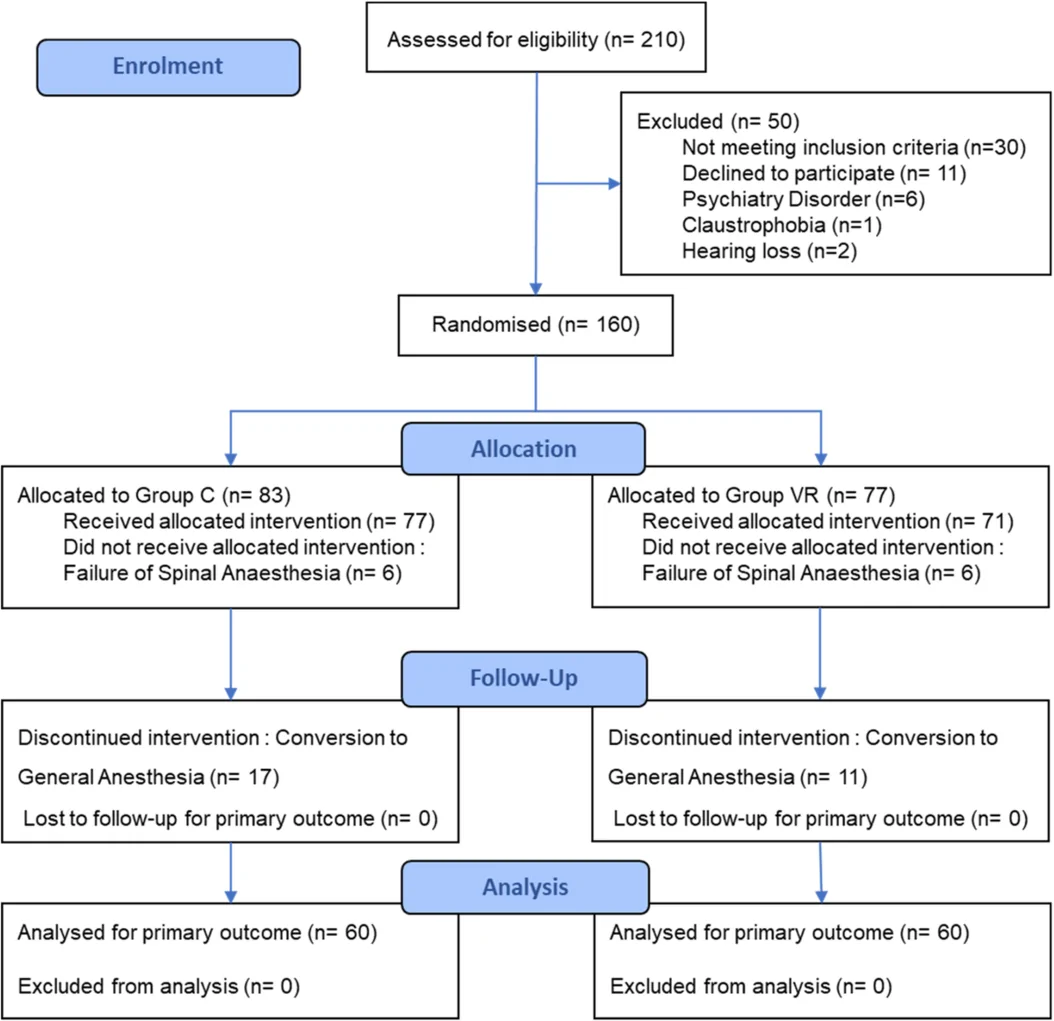
Flow chart of the study population.

A total of 210 patients were assessed for eligibility, of whom 50 were excluded (not meeting inclusion criteria, *n* = 30; declined to participate, *n* = 11; psychiatric disorder, *n* = 6; claustrophobia, *n* = 1; hearing loss, *n* = 2). The remaining 160 patients were randomized into two groups: the standard care group (Group C, *n* = 83) and the VR group (Group VR, *n* = 77). In Group C, 6 patients did not receive the allocated intervention due to failure of spinal anesthesia, and a further 17 discontinued the intervention due to conversion to general anesthesia, resulting in 60 patients analyzed for the primary outcome. In Group VR, 6 patients did not receive the allocated intervention due to failure of spinal anesthesia, and a further 11 discontinued due to conversion to general anesthesia, resulting in 60 patients analyzed for the primary outcome. No patients were lost to follow‐up for the primary outcome, and none were excluded from the analysis in either group (Figure 4).

### 3.1 General Characteristics

The socio‐demographic, anthropometric, lifestyle, and clinical characteristics were comparable in the two groups (all *p* > 0.05) (Table 1).

**Table 1:** Baseline socio‐demographic and anthropometric characteristics.

			Group C *N* (%)	Group VR *N* (%)	*p* value
Socio‐demographic characteristics	Age (years) (median [IQR])		34.4 [23.4–49.4]	41 [28–50]	0.338***
	Gender	Male	27 (45)	25 (41.7)	0.713*
		Female	33 (55)	35 (58.3)	
	Education levels	Illiterate	14 (23.3)	12 (20)	0.658*
		Primary	12 (20)	15 (25)	0.512*
		Secondary	14 (23.3)	16 (26.7)	0.673*
		University	20 (33.3)	17 (28.3)	0.533*
	Marital status	Single	31 (51.7)	25 (41.7)	0.272*
		Married	23 (38.3)	29 (48.3)	0.269*
		Divorced	4 (6.7)	1 (1.7)	0.364*
		Widowed	2 (3.3)	5 (8.3)	0.439*
Anthropometric characteristics	Weight (kg) (median [IQR])		77.5 [66.1–83.7]	74.3 [67.1–87.8]	0.637***
	Height (cm) (median [IQR])		171.5 [167.1–177.6]	171.5 [163.3–178.5]	0.741***
	BMI (kg/m^2^) (median [IQR])		25.3 [21.4–28.4]	24.9 [21.8–28.9]	0.832***

Abbreviations: ASA, American Society of Anesthesiologists; BMI, Body mass index; Group C, group control; Group VR, group virtual reality; IQR: interquartile range.

* Chi square Test.

**Fisher exact Test.

*** Mann‐Whitney Test; *p* value ≤ 0.05: level of significance.

### 3.2 Perioperative and Surgery Characteristics

Surgical procedures and perioperative timings were comparable between groups (*p *> 0.05). Tibial fractures were most common (25% vs. 30%), followed by ankle fractures (33.3% vs. 31.7%). No significant differences existed in OR entry time, block onset, surgery duration, or recovery times (Table 2).

**Table 2:** Perioperative characteristics and duration of surgery.

			Group C	Group VR	*p* value
APAIS	Need for information	Score (median [IQR])	6 [5–7]	6 [5–7]	0.200***
		Low	6 (10)	3 (5)	0.491*
		Moderate	47 (78.3)	46 (76.7)	0.827*
		High	7 (11.7)	11 (18.3)	0.306*
	Anxiety	Score (median [IQR])	12 [10.5–13]	11.5 [11–13]	0.320***
		Low level	15 (25)	13 (21.7)	0.666*
		High level	45 (75)	47 (78.3)	
			**Median [IQR]**	**Median [IQR]**
Duration: Incision → Closure (min)			111 [105–116]	109 [104–113]	0.264***
Duration in OU: Entry → Exit (min)			190 [175–204]	226 [198–250]	< 0.001***

Abbreviations: Anxiety‐VAS, Anxiety‐Visual Analog Scale; Group C, group control; Group VR, group virtual reality; IQR: interquartile range; OU, operating unit; OR, operating room.

* Chi square Test.

**Fisher exact Test.

*** Mann‐Whitney Test; *p* value ≤ 0.05: level of significance.

However, a significant difference was observed in the overall duration of stay in the operating unit (OU), which was longer in the VR group compared with the control group (226 [198–250] min vs. 190 [175–204] min, *p* < 0.001).

### 3.3 Perioperative Anxiety Using STAI

The STAI‐6 scores showed no significant difference between groups at baseline (preoperative assessment), with Group C scoring 48.22 ± 9.13 and Group VR scoring 48.94 ± 9.38 (*p* = 0.729). However, postoperative assessment (T3) revealed a significant reduction in anxiety levels in the virtual reality group compared to the control group. Group C maintained relatively stable anxiety scores at 46.72 ± 2.58, while Group VR demonstrated a marked decrease to 36.50 ± 7.92 (*p* < 0.001). When comparing preoperative to postoperative changes within each group, Group C showed no significant change in STAI‐6 scores (*p* = 0.305), whereas Group VR exhibited a statistically significant reduction in anxiety levels from baseline to postoperative assessment (*p* < 0.001).

A two‐way repeated measures ANOVA was conducted to examine the effect of group (VR vs. Control) and time (preoperative vs. postoperative) on STAI‐6 scores. The analysis revealed a significant main effect of time (F(1, 118) = 45.20, *p* < 0.001, partial η^2^ = 0.277), indicating that anxiety scores decreased significantly from preoperative (M = 48.58, SD = 9.22) to postoperative assessment (M = 41.61, SD = 9.69). A significant main effect of group was also found (F(1,118) = 15.16, *p* < 0.001, partial η^2^ = 0.114), with the VR group showing lower overall anxiety scores (M = 42.72) compared to the control group (M = 47.47). Most importantly, a significant time × group interaction was observed (F(1, 118) = 27.84, *p* < 0.001, partial η^2^ = 0.191), indicating that the reduction in anxiety over time differed significantly between groups. Post‐hoc pairwise comparisons revealed that while both groups had comparable preoperative scores (VR: 48.94 vs. Control: 48.22), the VR group demonstrated a significantly greater reduction in anxiety postoperatively (36.5) compared to the control group (46.72), with a mean difference of 4.75 points (*p* < 0.001, 95% CI [2.33, 7.17]).

### 3.4 Perioperative Anxiety Using APAIS

The APAIS showed comparable overall scores, with the majority of participants in both groups reporting moderate information needs. The anxiety subscale scores were similar between groups (Table 2).

### 3.5 Perioperative Anxiety Using VAS

Preoperative Anxiety‐VAS showed no significant differences between groups; however, postoperative Anxiety‐VAS revealed a significant reduction in intense anxiety in the VR group, with 50% of Group C reporting intense anxiety compared to only 25% in Group VR (*p *= 0.005) (Table 3).

**Table 3:** Anxiety and Pain results of the study population.

		Preoperative (T0)		*p*‐value	Postoperative (T3)		*p* value
		Group C	Group VR		Group C	Group VR	
Anxiety‐VAS	– Absence	7 (11.7)	5 (8.3)	0.543*	6 (10)	17 (28.3)	0.094**
	– Mild to moderate	30 (50)	35 (58.3)	0.360*	24 (40)	28 (46.7)	0.461*
	– Intense	23 (38.3)	20 (33.3)	0.100*	30 (50)	15 (25)	**0.005** *
Pain‐VAS	– Absence	10 (16.7)	8 (13.3)	0.609*	7 (11.7)	6 (10)	0.769*
	– Mild to moderate	27 (45)	28 (46.7)	0.855*	28 (46.7)	27 (45)	0.855*
	– Intense	23 (38.3)	24 (40)	0.852*	25 (41.7)	27 (45)	0.713*

Abbreviations: Group C, group control; Group VR, group virtual reality; IQR, interquartile range; Pain‐VAS, Pain‐Visual Analog Scale; SD, standard deviation.

* Chi square Test.

** Fisher exact Test; *p* value ≤ 0.05: level of significance.

A two‐way repeated measures ANOVA was conducted to examine the effect of group (VR vs. Control) and time (T0 to T6) on Anxiety‐VAS scores. Mauchly's test indicated that the assumption of sphericity was violated (χ^2^ = 146.63, *p* < 0.001); therefore, Greenhouse‐Geisser corrections were applied. The analysis revealed no significant main effect of time (F(4.27, 503.33) = 1.15, *p* = 0.331, partial η^2^ = 0.010), indicating that anxiety scores did not change significantly across time points when groups were combined. However, a significant main effect of group was found (F(1, 118) = 26.20, *p* < 0.001, partial η^2^ = 0.182), with the VR group demonstrating lower overall anxiety scores (M = 4.21) compared to the control group (M = 5.35). Most importantly, a significant time × group interaction was observed (F(4.27, 503.33) = 6.10, *p* < 0.001, partial η^2^ = 0.049), indicating that the pattern of anxiety changes over time differed significantly between groups.

Post‐hoc analysis revealed that anxiety significantly increased from T0 (before OR) to T2 (at OR before spinal anesthesia) by 0.65 points (*p* = 0.040), then significantly decreased from T2 to T4 (at H2) by 0.69 points (*p* = 0.039). While both groups had comparable anxiety levels at baseline and during the early perioperative period (T0‐T2), the VR group showed significantly lower anxiety scores from T3 onwards through postoperative recovery (after incision: VR = 3.87 vs. Control = 5.67; at H2: VR = 3.37 vs. Control = 5.57; at H4: VR = 3.87 vs. Control = 5.73; at H6: VR = 3.93 vs. Control = 5.83), with mean differences ranging from 1.70 to 2.20 points. These findings indicate sustained anxiolytic effects of VR intervention throughout the surgical and immediate postoperative periods.

### 3.6 Pain

Pain‐VAS scores at baseline (T0) were comparable across all intensity levels (*p *> 0.05), and postoperative Pain‐VAS (T3) remained similar between groups with no significant differences observed (Table 3).

### 3.7 Perioperative Evolution

**Figure 5:**
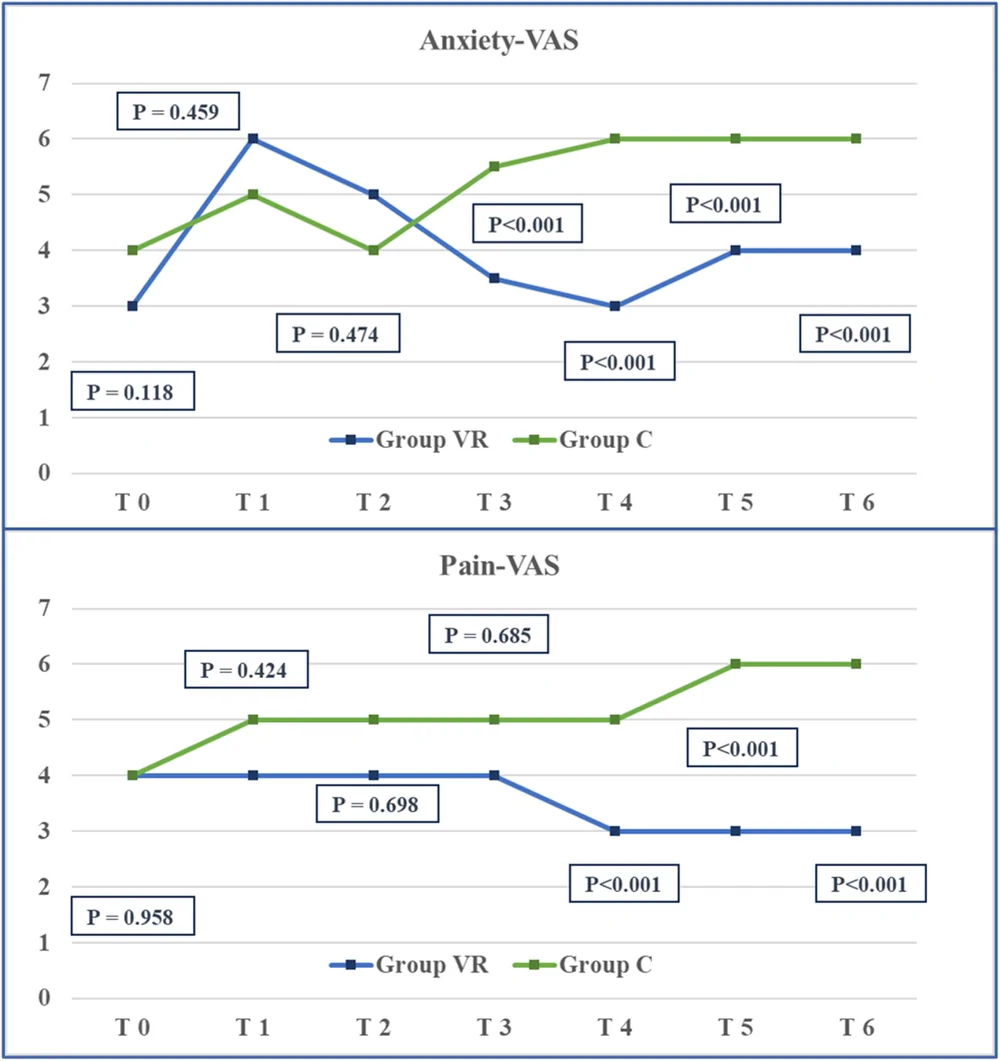
Perioperative Data of the study population.

During intraoperative to postoperative period (T3‐T6), VR patients had significantly lower anxiety scores compared to controls (*p *< 0.001). From T4 onwards, VR patients also reported lower pain scores (*p *< 0.001) (Figure 5). Hemodynamic parameters (blood pressure and heart rate) remained stable and comparable between groups throughout the procedure.

### 3.8 Perioperative Outcomes

VR patients required significantly less midazolam (20% vs. 53.3%, *p* = 0.001), suggesting reduced need for anxiolytic medication. Intraoperative and postoperative systemic opioid consumption, expressed as intravenous morphine milligram equivalents (MME), was significantly lower in the VR group compared with the control group (12 ± 5.4 vs. 22.5 ± 7.3 mg MME, *p* < 0.001). This suggests VR's anxiolytic effect reduced opioid requirements rather than compensatory increases in analgesic administration.

Ephedrine use showed no significant difference (15% vs. 23.3%, *p* = 0.246), indicating comparable hemodynamic stability. Postoperative analgesic use was similar between groups (38.3% vs. 40%, *p* = 0.852), though paracetamol was more frequent in VR patients while tramadol was more common in controls (differences not significant) (Table 4).

**Table 4:** Perioperative outcomes of the study population.

		Group CN (%)	Group VRN (%)	*p* value
Use of midazolam	Yes	32 (53.3)	12 (20)	**0.001** *
	No	28 (46.7)	48 (80)	
Use of ephedrine	Yes	14 (23.3)	9 (15)	0.246*
	No	46 (76.7)	51 (85)	
Postoperative use of analgesics	Yes	24 (40)	23 (38.3)	0.852*
	No	36 (60)	37 (61.7)	
Type of analgesics	Paracetamol	11 (18.3)	17 (28.3)	0.195*
	Tramadol	13 (21.6)	6 (10)	0.073*
Postoperative nausea	Yes	22 (36.7)	11 (18.3)	**0.025** *
	No	38 (63.3)	49 (81.7)	
Postoperative vomiting	Yes	13 (21.7)	8 (13.3)	0.230**
	No	47 (78.3)	52 (86.7)	
Postoperative headache	Yes	21 (35)	10 (16.7)	**0.002** *
	No	39 (65)	50 (83.3)	
Satisfaction of patients	Extremely dissatisfied	2 (3.3)	1 (1.7)	1**
	Dissatisfied	26 (43.3)	2 (3.3)	**< 0.001** *
	Undecided	22 (36.7)	8 (13.3)	**0.003** *
	Satisfied	7 (11.7)	28 (46.7)	**< 0.001** *
	Extremely satisfied	3 (5)	21 (35)	**< 0.001** *
Satisfaction of anesthesia team	Extremely dissatisfied	2 (3.3)	1 (1.7)	1**
	Dissatisfied	27 (45)	4 (6.7)	**< 0.001** *
	Undecided	16 (26.7)	11 (18.3)	0.274*
	Satisfied	6 (10)	21 (35)	**0.001** *
	Extremely satisfied	9 (15)	23 (38.3)	**0.004** *
Satisfaction of surgical team	Extremely dissatisfied	3 (5)	1 (1.7)	0.619*
	Dissatisfied	24 (40)	2 (3.3)	**< 0.001** *
	Undecided	22 (36.7)	20 (33.3)	0.702*
	Satisfied	9 (15)	24 (40)	**0.002** *
	Extremely satisfied	2 (3.3)	13 (21.7)	**0.002** *

Abbreviations: Group C: group control; Group VR: group virtual reality.

* Chi square Test.

** Fisher exact Test; *p* value ≤ 0.05: level of significance.

The VR group experienced fewer postoperative complications. Nausea was significantly reduced (18.3% vs. 36.7%, *p* = 0.025), and headache was markedly lower (16.7% vs. 35%, *p* = 0.002). Postoperative vomiting, while less frequent in the VR group (13.3% vs. 21.7%), did not reach statistical significance (*p *= 0.230) (Table 4).

No cybersickness was observed in any patient in the VR group during VR exposure.

### 3.9 Satisfaction

Patient satisfaction differed notably between groups. Dissatisfaction was reported in 43.3% of controls versus 3.3% of VR patients (*p* < 0.001). Satisfaction was observed in 46.7% of VR patients compared with 11.7% of controls (*p* < 0.001), while extreme satisfaction was reported by 35% of VR patients and 5% of controls (*p* < 0.001).

For the anesthesia team, dissatisfaction was noted in 45% of controls and 6.7% of VR cases (*p* < 0.001). Satisfaction was documented in 35% of VR cases and 10% of controls (*p* = 0.001), and extreme satisfaction in 38.3% versus 15% (*p* = 0.004).

For the surgical team, dissatisfaction was observed in 40% of controls and 3.3% of VR cases (*p *< 0.001). Satisfaction was reported in 40% of VR cases and 15% of controls (*p *= 0.002), while extreme satisfaction was recorded in 21.7% and 3.3%, respectively (*p *= 0.002) (Table 4).

## 4 Discussion

This randomized controlled trial demonstrates that VR distraction significantly improves perioperative anxiety management and reduces sedative medication requirements in patients undergoing lower limb orthopedic surgery under spinal anesthesia. These findings add novel evidence to the growing literature supporting VR as an effective non‐pharmacological adjunct in perioperative care.

### 4.1 Anxiety Reduction and Patient Experience

The immersive nature of VR created a sense of presence that effectively isolated patients from anxiety‐provoking stimuli in the OR environment, including visual cues such as surgical equipment and auditory stimuli such as medical staff conversations [5, 6]. This sensory isolation is not achievable with conventional video viewing on a flat screen or audio listening, as patients remain visually and psychologically anchored in the surgical setting [7, 11].

Furthermore, the interactive and engaging nature of the VR content required sustained attentional resources, modulating cognitive load in a way that limited patients' capacity to focus on anxiety‐related thoughts and perioperative concerns. This mechanism of cognitive load modulation through immersive engagement differs fundamentally from passive distraction techniques, which do not demand the same level of cognitive investment or provide the same degree of psychological separation from the physical environment.

A key finding of this trial is the significant reduction in perioperative anxiety with VR, as reflected by both categorical anxiety levels and STAI‐6 scores. Our study extends previous knowledge by showing that VR's anxiolytic effects persisted into the postoperative period, suggesting durable psychological benefit rather than a transient distraction effect. This sustained reduction in anxiety has implications for improved recovery and enhanced patient experience. This effect is consistent with recent meta‐analyses and clinical trials reporting VR's anxiolytic benefits in surgical settings [7, 13, 14].

The sustained anxiolytic effects observed from T3 through T6 (spanning intraoperative through postoperative periods) suggest that VR's impact extends beyond momentary distraction, potentially reflecting neurophysiological changes associated with immersive experience, such as modulation of autonomic nervous system activity and stress hormone release. The sense of control and agency provided by interactive VR may also have contributed to enhanced psychological resilience during the surgical experience, a benefit not available through passive audiovisual content consumption. Preoperative STAI‐6 scores were numerically, though not statistically, higher in the VR group (48.94 ± 9.38 vs. 48.22 ± 9.13, *p* = 0.729). We interpret this as a chance imbalance arising from randomization in a modestly sized sample rather than a true baseline difference, and possibly reflecting anticipatory apprehension related to unfamiliarity with the VR equipment itself before its anxiolytic benefit could be experienced. Because groups were statistically equivalent at baseline, this small numerical difference does not affect interpretation of the treatment effect.

### 4.2 Pain and Analgesic Use

While immediate postoperative pain scores did not differ significantly between groups, VR appeared to influence pain perception later in recovery (T4–T6), aligning with other randomized controlled trials in spinal anesthesia settings [15, 16, 17]. The absence of differences in postoperative analgesic consumption indicates that VR primarily modulates the emotional component of pain through distraction and cognitive engagement rather than direct nociceptive pathways [10]. This highlights VR's potential role as a supportive strategy for anxiety‐driven pain amplification rather than a replacement for pharmacological analgesia.

### 4.3 Reduction in Sedative Requirements

One of the most clinically relevant observations is the 63% reduction in midazolam use in the VR group, with only 20% of VR patients requiring midazolam compared to 53.3% in the control group (*p* = 0.001). This finding is particularly significant in the context of spinal anesthesia, where oversedation can compromise respiratory safety. Our results, in line with prior reports [3, 6, 13], suggest VR can provide adequate anxiolysis while minimizing sedative exposure — potentially translating into fewer drug‐related complications, faster recovery, and reduced healthcare costs.

### 4.4 Hemodynamic Stability and Safety

VR use was associated with stable hemodynamic parameters, with no excess requirement for vasopressors. These findings corroborate VR's safety in the perioperative setting [10], and support its integration into anesthetic practice without concern for cardiovascular compromise.

### 4.5 Postoperative Complications and Recovery

Our study revealed reduced rates of postoperative nausea and headache in the VR group. Nausea was significantly reduced, and headache was markedly lower. Postoperative vomiting, while numerically less frequent in the VR group, did not reach statistical significance. These improvements may reflect the indirect benefits of reduced anxiety and sedative use. The decrease in post‐dural puncture headache is an original finding in our study population and warrants further investigation, as the mechanism remains speculative but may involve reduced anxiety‐related muscle tension or improved cooperation during positioning [6, 14].

### 4.6 Satisfaction Among Patients and Clinicians

The consistently higher satisfaction scores among patients, anesthesiologists, and surgeons reinforce the acceptability of VR in clinical practice. Similar benefits have been reported in other surgical specialties [3, 18, 19]. This multi‐stakeholder acceptance is critical for implementation, as it facilitates smoother workflows and enhances overall perioperative care.

### 4.7 Clinical and Economic Implications

Although VR use was associated with a slightly longer OU stay, this should be interpreted in the context of equipment installation time, which can be reduced with increased team experience.

These benefits should be weighed against the modestly longer OU stay, though future research incorporating formal cost‐effectiveness analyses is needed to quantify VR's net economic value.

### 4.8 Strengths and Limitations

This randomized controlled trial is among the few to assess VR distraction during lower limb orthopedic surgery under spinal anesthesia. Its strengths include a relatively large sample, a standardized anesthetic protocol, and the use of validated anxiety measures, which enhance the reliability of findings.

However, several limitations should be noted. The single‐center design may limit generalizability, and complete blinding was not possible, introducing potential bias. Long‐term outcomes and cost‐effectiveness were not assessed, and the slightly longer OU stay warrants further evaluation. The final sample size (*n* = 120) exceeded the a priori calculated minimum (*n* = 64); while this reflected planned considerations around the recruitment window, we note that pre‐specifying a single combined justification at the protocol stage, rather than multiple concurrent rationales, would strengthen the transparency of similar future trial designs.

Future multicenter trials with economic and long‐term outcome analyses, as well as comparisons of different VR content and delivery methods, are needed to optimize and broaden the use of VR in perioperative care.

## 5 Conclusion

This randomized controlled trial provides robust evidence that VR distraction is an effective, non‐pharmacological adjunct to standard care during lower limb orthopedic surgery under spinal anesthesia, significantly reducing perioperative anxiety, sedative and opioid requirements, and postoperative complications while improving satisfaction across patients, anesthesia, and surgical teams. Using commercially available, scalable equipment, VR technology can be readily integrated into routine perioperative care protocols, supporting medication stewardship across diverse clinical settings, with future studies extending this to elderly or medically complex patients beyond our age‐restricted cohort.

## Author Contributions

**Fedi Ben Dhaou:** conceptualization, investigation, funding acquisition, writing – original draft, writing – review and editing, visualization, methodology, validation, formal analysis, software, project administration, resources, supervision, data curation. **Yosra Mejdoub:** methodology, validation, project administration, supervision, resources, writing – review and editing, funding acquisition. **Maroua Trigui:** formal analysis, funding acquisition, methodology, resources, software, validation, visualization, writing – original draft, writing – review and editing. **Emna Mziou:** formal analysis, funding acquisition, methodology, resources, software, validation, visualization, writing. **Amal Ayedi:** data curation, funding acquisition, investigation, methodology, software, visualization, writing – original draft, writing – review and editing. **Meriam Boussarsar:** data curation, funding acquisition, resources, investigation, supervision, visualization, writing – review and editing. **Mariem Keskes:** data curation, funding acquisition, resources, investigation, validation, visualization, writing – review and editing. **Yassine Maktouf:** data curation, funding acquisition, resources, investigation, supervision, visualization, writing – review and editing. **Fares Mezghani:** data curation, funding acquisition, resources, investigation, supervision, visualization, writing – review and editing. **Mohamed Seddik Regaieg:** data curation, funding acquisition, resources, investigation, supervision, visualization, writing – review and editing. **Tarek Bardaa:** funding acquisition, investigation, methodology, resources, supervision, validation, visualization, writing – review and editing. **Chams Elhouda Ben Dhaou:** funding acquisition, validation, visualization, writing – original draft, writing – review and editing. **Hichem Cheikhrouhou:** conceptualization, funding acquisition, resources, methodology, project administration, supervision, validation, visualization, writing – review and editing. **Imen Zouche:** methodology, validation, supervision, project administration, resources, writing – review and editing, funding acquisition.

## Funding

This research was self‐funded by the authors. No external financial support was received for the conduct, authorship, or publication of this study. As the study was self‐funded, no external funding source had any role in the study design; the collection, analysis, or interpretation of data; the writing of this report; or the decision to submit the report for publication.

## Ethics Statement

This study was approved by the Ethics Committee of the Faculty of Medicine of the University of Sfax and the Direction of Habib Bourguiba University Hospital, Sfax, Tunisia (approval reference: 51/24), and was conducted in accordance with the ethical standards of the Declaration of Helsinki and its later amendments. The trial was prospectively registered with the Pan African Clinical Trial Registry (PACTR202507660086282) and ClinicalTrials.gov (NCT07134764).

## Consent

Informed consent was obtained from all individual participants included in the study, after they received a detailed explanation of the study objectives, research modalities, and implications of participation. Consent was also obtained for the anonymized use and publication of study data.

## Conflicts of Interest

The authors declare no conflicts of interest.

## Author Approval and Data Access Statement

All authors have read and approved the final version of the manuscript. Fedi Ben Dhaou, the corresponding author and manuscript guarantor, had full access to all of the data in this study and takes complete responsibility for the integrity of the data and the accuracy of the data analysis.

## Use of Artificial Intelligence

An AI‐based language‐editing tool was used solely to assist with drafting and improving the readability of the manuscript text. AI was not used for study design, data collection, data analysis, data interpretation, or generation of results. All scientific content was reviewed, verified, and approved by the authors.

## Transparency Statement

The lead author, Fedi Ben Dhaou, affirms that this manuscript is an honest, accurate, and transparent account of the study being reported; that no important aspects of the study have been omitted; and that any discrepancies from the study as planned (and registered) have been explained.

## Supporting information


Supporting File 1

Supporting File 2

## Data Availability

The data that support the findings of this study are available from the corresponding author upon reasonable request. The datasets generated and/or analyzed during the current study are available from the corresponding author upon reasonable request with the authorization of all authors.
