# Supplementary material for: Virtual Reality for Anxiety and Pain Management in Lower Limb Orthopedic Surgery Under Spinal Anesthesia: A Randomized Controlled Trial
Source: Health Sci Rep. 2026 Sep 24;9(10):e73146. doi: 10.1002/hsr2.73146 (PMC13602353; doi:10.1002/hsr2.73146)
Supplement: Supplementary file 1 [file HSR2-9-e73146-s002.docx]

**Satisfaction evaluation criteria**
